# Measurement properties of the Short Form-36 (SF-36) and the Functional Assessment of Cancer Therapy - Anemia (FACT-An) in patients with anemia associated with chronic kidney disease

**DOI:** 10.1186/s12955-018-0933-8

**Published:** 2018-05-31

**Authors:** Fredric O. Finkelstein, Floortje van Nooten, Ingela Wiklund, Dylan Trundell, David Cella

**Affiliations:** 1 Yale University, New Haven, CT USA; 2 Formerly with Astellas Pharma BV, Sylviusweg 62, 2333 BE Leiden, The Netherlands; 3 Evidera, Metro Building, 6th Floor, 1 Butterwick, London, W6 8DL UK; 4 Formerly with Evidera, Metro Building, 6th Floor, 1 Butterwick, London, W6 8DL UK; 5 Department of Medical Social Sciences, Northwestern University, Evanston, IL USA

**Keywords:** Anemia, Chronic kidney disease, Quality of life, Psychometric evaluation, SF-36, FACT-An

## Abstract

**Background:**

Anemia is a common and debilitating manifestation of chronic kidney disease (CKD). Data from two clinical trials in patients with anemia of CKD were used to assess the measurement properties of the Medical Outcomes Survey Short Form-36 version 2 (hereafter SF-36) and the Functional Assessment of Cancer Therapy-Anemia (FACT-An). The Vitality and Physical functioning domains of the SF-36 and the FACT-An Total, Fatigue and Anemia subscales were identified as domains relevant to CKD-associated anemia.

**Methods:**

A total of 204 patients aged 18–80 years were included in the analyses that included internal consistency (Cronbach’s alpha), test-retest reliability (intraclass correlation coefficients [ICCs]), convergent and known-groups validity, responsiveness, and estimates of important change.

**Results:**

Both the SF-36 and the FACT-An had strong psychometric properties with high internal consistency (Cronbach’s alpha: 0.69–0.93 and 0.79–0.95), and test-retest reliability (ICCs: 0.64–0.83 and 0.72–0.88). Convergent validity, measured by correlation coefficients between similar concepts in SF-36 and FACT-An, ranged from 0.52 to 0.77. Correlations with hemoglobin (Hb) levels were modest at baseline; by Week 9, the correlations with Hb were somewhat higher, *r =* 0.23 (*p* < 0.05) for SF-36 Vitality, *r* = 0.22 (*p* < 0.05) for FACT-An Total, *r* = 0.26 (*p* < 0.001) for FACT-Fatigue and *r =* 0.22 (*p* < 0.01) for Anemia. Correlations with Hb at Week 13/17 were *r* = 0.28 (*p* < 0.001) for SF-36 Vitality and *r* = 0.25 (*p* < 0.05) for Role Physical; FACT-An Total correlation was *r* = 0.33 (*p* < 0.0001), Anemia was *r* = 0.28 (p < 0.001), and Fatigue was *r* = 0.30 (*p* < 0.001).

The SF-36 domains and Component Summary scores (*p* < 0.05–*p* < 0.0001) demonstrated ability to detect change. For the FACT-An, significant differences (*p* < 0.05–*p* < 0.0001) were observed between responder and non-responder change scores: important change score estimates ranged from 2 to 4 for Vitality and 2–3 for Physical functioning. Important change scores were also estimated for the FACT-An Total score (6–9), the Anemia (3–5), and Fatigue subscale (2–4).

**Conclusions:**

Both the SF-36 Vitality and Physical function scales and the FACT-An Total, Fatigue and Anemia scales, are reliable and valid measures for assessing health-related quality of life in anemia associated with CKD.

## Background

Anemia is common among patients with chronic kidney disease (CKD); the prevalence in US patients is estimated to be over 15% [1]. An association between anemia and CKD stage has been identified, with anemia increasing in prevalence by disease severity from 8.4% in Stage 1 to 53.4% in Stage 5. Minutolo et al. [2] reported a 44% prevalence of anemia across all CKD stages in patients who were not receiving dialysis, while McFarlane et al. estimated a prevalence of 50 to 70% across all stages [3].

Symptoms of anemia include fatigue, low energy, weakness, dizziness, and dyspnea [4, 5]; symptom severıty varies wıth the degree of anemia [6]. Persistent fatigue has been identified as one of the most debilitating symptoms both pre-dialysis and in dialysis disease stages [7, 8]. The impact of fatigue varies widely, from being described as ‘mild impairment’ to greatly affecting daily functioning including, at times, basic activities of daily living [9]. Diminished aspects of health-related quality of life (HRQoL), such as functional and ambulatory impairment, and increased risk of falls, have all been identified as complications of anemia [10–14].

Patient-reported outcome (PRO) measures are frequently used to assess treatment efficacy in clinical trials [15]. Although used in some trials as primary endpoints (eg, a patient report of pain is required in the absence of objective clinical markers), PROs are often used to identify additional benefits associated with treatment, including symptom experience, functioning, and HRQoL. Thus, measurement of HRQoL as a primary outcome of treatment interventions in end-stage renal disease (ESRD), as well as a tool for clinicians to assess patient status, is an increasingly accepted research endpoint.

While earlier studıes suggested a broad range of symptom improvement wıth anemia treatment, recent studies indicate that the domains of Vitality and Physical functioning are most beneficially affected by treatment [6, 16].

The Medical Outcomes Survey Short Form-36 (SF-36) is a generic, widely validated HRQoL measure that has been used in numerous research studies and clinical trials of CKD anemia [11, 17–25] with specific focus on the Vitality and Physical functioning domains [6, 16, 26]. Measurement properties of SF-36 have been assessed in patients with CKD, [27] but no corresponding data are available specifically for patients with CKD anemia.

The Functional Assessment of Cancer Therapy-Anemia (FACT-An) was developed to assess the impact of anemia on quality of life in patients with cancer-associated anemia [28, 29]. Content validity was addressed during the development of the FACT-An and was based on interviews with patients with cancer-associated anemia, literature review, and expert input. The Total FACT-An score and the FACT Anemia and Fatigue score are of particular interest for use with anemia associated with CKD. Although the tool has been used in many oncology clinical trials, [30–33], data in the CKD population are lacking.

Both the European Medicines Agency [34] and the US Food and Drug Administration [35] require that new drugs under consideration for approval be tested in clinical trials with PRO endpoints that are specific and relevant to the proposed treatment population. Documentation of measurement properties of domains of PRO surveys in the target population is essential. The purpose of this study is to assess further the measurement properties of the SF-36 and the FACT-An with particular focus on domains of vitality/fatigue, anemia, and physical function, which most closely relate to CKD anemia. Data from two clinical trials in patients with CKD anemia who were not receiving dialysis or who were newly initiated on dialysis were used to evaluate the validity of SF-36 and the FACT-An in this sample.

## Methods

### Patient sample

Data were derived from two clinical trials that evaluated a hypoxia-inducing factor prolyl hydroxylase (HIF-PH) inhibitor in patients with CKD anemia. The trial designs were similar, but not identical, and allow the evaluation of the SF-36 and FACT-An questionnaires in patients with ESRD. Details of these trials including treatment interventions have been published previously [36–38]. Briefly, the first (NCT00761657) was a Phase 2, open-label, randomized trial in patients with anemia and Stage 3 or 4 CKD, who were not on dialysis (non-dialysis group) [36]. The patients were 18–70 years old with an Hb level < 10.5 g/dL and an estimated glomerular filtration rate of > 15 to < 60 mL/min/1.73 m^2^ pre-randomization. The second (NCT01244763) was a Phase 2b, randomized, open-label trial in newly initiated patients on dialysis (dialysis group) [37, 38]. The patients were 18–80 years old with a pre-randomization Hb level < 10.0 g/dL. They had received hemodialysis or peritoneal dialysis for native kidney ESRD for a minimum of 2 weeks and a maximum of 4 months. Patients in both studies were not currently and had not previously received an erythropoietin-stimulating agent (ESA) or IV iron within 4 weeks of randomization.

### Study design

In the non-dialysis trial, dosing strategies for the HIF-PH inhibitor were employed across six cohorts (with 24–25 patients per cohort). Patients attended weekly study visits during the treatment period (16 weeks for Cohorts A and B, and 24 weeks for Cohorts C through F). The FACT-An and the SF-36 questionnaires were administered at baseline, Week 9, Week 17 (Cohorts A–D only for SF-36), and Week 24 (Cohorts C–D only).

In the dialysis trial, approximately 12 patients were enrolled in each of five HIF-PH inhibitor treatment arms. The trial employed a 1:1:1 ratio for the first 36 patients to receive no iron supplementation, oral iron supplementation, and IV iron supplementation. Patients visited the clinic weekly during the 12-week treatment period, followed by a four-week post-treatment follow-up period. The FACT-An and SF-36 questionnaires were administered at baseline, Week 9, and Week 13.

Data used to characterize each patient sample were collected at baseline/during screening for both dialysis and non-dialysis patients. These included sociodemographic (date of birth, sex, and race/ethnicity) and clinical data (weight and height, pulse rate, blood pressure, respiratory rate, date of CKD diagnosis, date of anemia diagnosis, comorbid conditions, laboratory parameters, treatments received, and rescue medication use). Hb level was collected at baseline, Week 9, and Week 13/17 (dialysis/non-dialysis) as well as at other time points.

### Patient-reported outcome measures

The SF-36 is designed to assess health concepts that are relevant across age, disease, and treatment groups in adults [39]. As well as a health transition item, the SF-36 contains eight domains: Physical functioning (10 items), Role-physical (4 items), Bodily Pain (2 items), General Health (5 items), Vitality (4 items), Social functioning (2 items), Role-emotional (3 items), and Mental Health (5 items). Two Component Summary scores, the Physical Component Summary (PCS) and the Mental Component Summary (MCS), can also be calculated with all scores using the norm-based approach (US population norm mean score for each domain and summary score is 50, with a standard deviation [SD] of 10) [40]. Anemia-related domains such as the Vitality and Physical functioning were previously identified to be of special interest [6, 16]. A four-week recall was used for the SF-36 in both trials. Suggested important change scores have been reported by the instrument developers for each domain and Component Summary score based on the 2009 US general population [40]. The suggested change scores are appropriate for *T*-scores ranging from 30 to 40. Where the *T*-score for the population exceeds this range, increasing the change score is recommended.

The FACT-An is designed to assess aspects of quality of life affected by anemia in patients with cancer [28]. Using a seven-day recall period, the 27 item FACT-General (FACT-G) includes four dimensions of well-being: Physical (7 items), Functional (7 items), Social/Family (SWB; 7 items), and Emotional (6 items). The FACT-An also includes 13 fatigue-specific items (the Fatigue Subscale) plus an additional 7 items specific to anemia and unrelated to fatigue. Anemia subscales such as FACT-An Total and the Fatigue and Anemia subscales were of special interest. These last 20 items (13 + 7) combine to form the Anemia subscale. Higher scores indicate better health status. An important change score estimate of 4 points for the Anemia total score and 3 points for the Fatigue score has previously been reported in patients with cancer [41]. In patients with CKD, a three-point or greater increase was previously identified as a clinically meaningful improvement on the FACT-Fatigue total score [42].

### Statistical analyses

Unless stated otherwise, data from the two clinical trials were pooled at baseline and at Week 9. Week 17 data from the non-dialysis group were pooled with Week 13 data from the dialysis group. Pooling the data from the two trials provided a sufficiently large sample size and a greater range of impairment within a CKD patient sample for more effective testing of the measurement properties of SF-36 and FACT-An. All statistical tests were two-sided and significance level was set at *p <* 0.05.

For the SF-36 domain/Component Summary scores and the FACT-An Total and subscale scores, descriptive statistics (mean, SD, median, and range) were calculated at baseline, Week 9, and Week 13/17.

### Reliability

Cronbach’s coefficient alpha was used to assess the internal consistency of the SF-36 domain scores and Component Summary scores and the FACT-An Total and relevant subscales at baseline. A Cronbach’s alpha ≥0.70 was considered an acceptable minimum value definition for good reliability [43]. Patterns of item-to-item correlations and item-to-total correlations, and the number of items in the subscale, were also considered. Alpha coefficients > 0.7 indicated good internal consistency, 0.4–< 0.69 moderate internal consistency, and < 0.4 low internal consistency reliability [44, 45].

Test-retest reliability was assessed in the subgroup of patients (*n* = 153) whose Hb level was classified as unchanged (average weekly Hb change within ±0.5 g/dL between Week 9 and Week 13/17 with no rescue therapy between these time points). The average weekly change in Hb was determined by comparing the weekly differences in Hb between Week 9 and every week from Week 10 to Week 13/17. Intraclass correlation coefficients (ICCs) were calculated to compare the SF-36 domain/Component Summary and the FACT-An Total and subscale scores at Week 9 and Week 13/17. An ICC ≥ 0.7 indicated good, 0.4–0.7 moderate, and < 0.4 poor test-retest reliability [44, 45].

### Validity

Validity refers to the extent to which an instrument measures what it is intended to measure; it is typically assessed by examining correlations with other indicators of similar/related constructs [46]. Correlations of 0.10 are considered small, correlations between 0.30 and 0.50 are regarded as moderate, and correlations of 0.50 or more are considered large [47]. Spearman’s rank-order correlation coefficients between the SF-36 domain/Component Summary scores and the FACT-An Total and subscale scores at baseline, Week 9, and Week 13/17 were used to assess convergent validity.

The SF-36 Vitality domain was previously identified as an important measure of disease impact resulting from anemia [6, 16], therefore the FACT-An Fatigue and Anemia subscales were expected to correlate more strongly with Vitality domain than other SF-36 domains. Known-groups validity was assessed to demonstrate the ability of the SF-36 and FACT-An instrument domain scores to differentiate between patients with varying levels of anemia, using Hb levels at baseline. The SF-36 and FACT-An domain scores were compared between the scores above and below the median Hb level at baseline using analysis of covariance (ANCOVA) with adjustments for gender and age. Similarly, for assessment of known groups validity, the SF-36 Physical Function and Vitality median split domain scores were used to establish the difference in the FACT-AN scores at baseline, while the FACT-An, FACT Anemia, and FACT Fatigue subscales median split were used to show that the baseline SF-36 scores differed. Groups were defined by Hb level (< 11 g/dL vs ≥ 11 dg/dL; < median vs ≥ median) or relevant SF-36 domain/ score and FACT-An specified domain score (< median vs ≥ median).

### Ability to detect change

The focus of this study was to document the ability of each instrument to detect change; thus, all analyses were conducted using pooled data only, i.e., no analyses were performed by treatment or doses. Using ANCOVA models, the ability to detect change of the SF-36 domain/Component Summary scores and the FACT-An Total and subscale scores was assessed by comparing the mean scores of Hb responders and non-responders at baseline to Week 9 and baseline to Week 13/17 change scores, controlling for age, gender, and baseline score (FACT-An and SF-36).

Responders were defined with the same clinical criteria used in the trial protocols:

- *Hb responders*
  Baseline Hb level > 8 g/dL: Hb level > 11.0 g/dL with ≥1.0 g/dL increase
  Baseline Hb level ≤ 8 g/dL: Hb level > 2.0 g/dL increase at the end of Weeks 7–9 and Weeks 11–13/15–17
- *SF-36 responders*
  *Vitality (SF-36) responders*: > 3-point increase in SF-36 Vitality score
  *Physical functioning (SF-36) responders*: > 3-point increase in SF-36 Physical functioning score
  *Physical Component Summary (SF-36) responders* -– an increase of two points in SF-36 Physical Component Summary score;
- *FACT-An responders*
  *FACT-An Anemia responders*: > 4-point increase in FACT-An Anemia score
  *FACT-An Fatigue responders*: > 3-point increase in FACT-An Fatigue subscale score.

### Estimating important change scores

Methods for interpreting the importance of quality of life changes in clinical research generally follow two approaches: distribution-based and anchor-based. A distribution-based approach is based on statistical characteristics of the obtained samples. This approach relies on the distribution of scores and the related effect size of change scores. An anchor-based approach compares the change in a patient reported outcome, such as patient judgement of change with a second, external measure of change, which serves as the anchor. Anchor-based assessments offer the advantage of linking the change in a given score to the patient’s perspective (which is captured by the anchor). Because we had relevant clinical anchors, we employed both approaches.

A minimally important difference (MID) refers to the “smallest difference in score in the domain of interest that patients perceive as important, (either beneficial or harmful, and which would lead the clinician to consider a change in the patient’s management” [48]. Because determination of the “minimally” important difference or change can vary by context, and because of some misuse of the concept, this term has fallen out of favor, with terms such as “important change scores” or “clinically important differences” increasingly used instead [49].

Previously reported important change scores for the SF-36 for the general population vary from 2 to 3 [40]. Clinically important differences reported for the FACT-An targeted domains have been estimated at 3 points for the Fatigue subscale, 4 FACT-An points for the Anemia subscale, and 6 points for the Total score [41].

In the present study, the SF-36 Vitality, Physical functioning domain, and Physical Component Summary score were used as anchors to estimate important change scores on the FACT-An. Similarly, FACT-An scores were used as anchors to estimate important change scores on the SF-36. Only patients achieving a pre-defined meaningful change score in these domains (from baseline to Week 9 or to Week 13/17) were included in the analyses. The mean SF-36 or FACT-An change scores (from baseline to Week 9 or to Week 13/17) for these patients thereby provided an estimate of an important change score. The meaningful change score was defined as 3–4 point increase in the SF-36 Vitality score, a 3–4 point increase in the SF-36 Physical functioning score, and a 2–3 point change in the SF-36 Physical Component Summary score.

For SF-36 change scores, the first anchor used a 4–7 point increase in the FACT-An Anemia subscale score to define the “minimally improved” category to assess the change in the SF-36 domain/Component Summary scores associated with a minimal change in condition. A second anchor used a 3–5 point increase in the FACT-An Fatigue subscale score.

The SF-36 Vitality domain (important change of 3 points) and Physical functioning domain (3 points) and the Physical Component Summary (2 points) were used to calculate important change score estimates for the FACT-An Total score and Fatigue and Anemia subscales. Similarly, the following FACT-An MID anchors [41] were used for SF-36 linking: FACT-An Total score of 6, FACT-An Anemia subscale score of 4, and a FACT-An Fatigue subscale score of 3 points.

## Results

### Patient demographics and clinical characteristics

The mean age of patients was 60.2 years (Table 1). The gender distribution differed by trial; 63.4% of patients in the non-dialysis group were female vs 47.5% in the dialysis group. Patients in the non-dialysis group were typically older than patients in the dialysis group. The statistically significant differences between the dialysis and non-dialysis groups should be interpreted with caution given the smaller number of patients in the dialysis group.

**Table 1:** Demographic Characteristics for Non-dialysis (NCT00761657) and Dialysis (NCT01244763) Patient Groups and Pooled Total

Characteristic	NCT00761657 (*N* = 145)	NCT01244763 (*N* = 59)	All Patients^a^(*N* = 204)
Age (years), mean (SD)***	64.4(11.3)	50.0(15.1)	60.2(14.0)
Gender (female), n (%)*	92(63.4%)	28(47.5%)	120(58.8%)
Race, n (%)**			
Asian	6(4.1%)	2(3.4%)	8(3.9%)
Black or African-American	42(29.0%)	2(3.4%)	44(21.6%)
White	92(63.4%)	54(91.5%)	146(71.6%)
Other^b^	5(3.4%)	1(1.7%)	6(2.9%)
Ethnicity (N, %)***			
Hispanic/Latino	47(32.4%)	1(1.7%)	48(23.5%)
Not Hispanic/Latino	98(67.6%)	58(98.3%)	156(76.5%)

* < 0.05, ** < 0.001, *** < 0.0001 comparing dialysis and non-dialysis patients

^a.^Subjects with at least one intake of study medication and at least one valid SF-36v2 completion

^b.^Other includes Mexican, mixed, Filipino, Spanish and Latin American

*SD* standard deviation

Comorbid conditions and disease history were generally similar; however, diabetes was more common in the non-dialysis group (61.4% vs 18.6%). A high proportion of the non-dialysis group had diabetic (55.9%) or hypertensive nephropathy (53.1%), whereas in the dialysis group, the most common CKD history category was ischemic nephropathy (42.4%). Additional details of the two patient groups can be found in respective trial publications [36, 38].

### Descriptive statistics

Low baseline SF-36 domain and Component Summary scores for the total sample were found for Physical functioning, Role-physical, General Health, and Physical Component Summary score (Table 2). Scores approaching the 2009 general US population mean (50.0) were found for Vitality, Mental Health, and Mental Component Summary score. The scores indicate that these patients predominantly experienced physical rather than mental impairments.

**Table 2:** Descriptive Statistics for the SF-36 and FACT-An at Baseline

Measure	N	Mean ± SD	Range	Median	Mode	Missing N, %
SF-36^a^						
Physical Functioning	194	38.2 ± 11.1	17.1–57.0	38.1	36.0	0 (0.0%)
Role-physical	194	40.1 ± 11.2	17.7–56.9	39.7	37.3	0 (0.0%)
Role-emotional	193	41.3 ± 13.7	9.2–55.9	44.2	55.9	1 (0.5%)
Vitality	194	47.7 ± 12.0	20.9–70.8	49.0	45.9	0 (0.0%)
Mental Health	194	48.4 ± 11.5	19.0–64.1	52.8	52.8	0 (0.0%)
Social Functioning	194	45.3 ± 11.5	13.2–56.9	45.9	56.9	0 (0.0%)
Bodily Pain	194	47.3 ± 11.2	19.9–62.1	46.1	62.1	0 (0.0%)
General Health	194	39.5 ± 9.5	16.2–62.5	38.6	32.9	0 (0.0%)
Physical Component Summary	194	40.0 ± 9.4	17.0–64.0	40.7	27.5	0 (0.0%)
Mental Component Summary	194	48.0 ± 11.5	14.3–67.3	49.6	32.1	0 (0.0%)
FACT-An						
Total FACT-An score	197	131.5 ± 30.0	44.0–185.0	131.0	122.0	1 (0.5%)
Total FACT-G score	197	77.6 ± 17.0	25.0–106.8	78.0	72.0	1 (0.5%)
Physical Well Being	198	20.9 ± 5.5	2.0–28.0	22.0	25.0	0 (0.0%)
Functional Well Being	197	18.0 ± 6.5	2.0–28.0	18.0	28.0	1 (0.5%)
Social Well Being	198	21.0 ± 5.8	2.0–28.0	22.0	28.0	0 (0.0%)
Emotional Well Being	197	17.8 ± 4.7	0.0–24.0	19.0	21.0	1 (0.5%)
Anemia subscale	198	53.9 ± 15.0	14.0–80.0	54.9	57.0	0 (0.0%)
Fatigue subscale	198	34.2 ± 11.5	8.0–52.0	35.5	28.0	0 (0.0%)
Trial outcome index	197	92.7 ± 24.6	27.0–136.0	92.0	72.0	1 (0.5%)

^a.^Norm-based score based on 2009 US general population survey

Baseline scores were similar for both trial samples for Physical functioning, Vitality, and the Physical Component Summary score, whereas Role-physical, Role-emotional, and Mental Health domains, and the Mental Component Summary score were all lower in dialysis patients.

At baseline, the mean FACT-An Total score was 131.5 (SD: 30.0), the mean Fatigue subscale score was 34.2 (SD: 11.5), and the mean Anemia subscale score was 53.9 (SD: 15.0). (Table 2) The FACT-An Well-Being subscales ranged from 17.8 to 21.0. Improvements were observed by Week 9 in the FACT-An Total and all subscale scores, except for the Social Well-Being where a small decrease was identified. These scores were relatively stable, with similar mean values reported at Week 13/17.

### Internal consistency and test-retest reliability

Good to excellent reliability coefficients were demonstrated for the SF-36 domain/Component Summary scores except the General Health Domain (0.69) and for the FACT-An Total score and all subscales (Table 3). Overall, Cronbach’s alpha scores were acceptable for both measures on all other domains, ranging from 0.76 to 0.95. Particularly high Cronbach’s alpha scores were observed in the Physical functioning, Role-physical, and Role-emotional domains on the SF-36 as well as the Anemia and Fatigue subscales on the FACT-An. Test-retest reliability was demonstrated for all domains and both summary scores, using > 0.6 as an acceptable cut-off [50].

**Table 3:** Internal Consistency and Test-retest Reliability of the SF-36 and the FACT-An Subscales

SF-36 domain/Component Summary score	N^a^	Cronbach’s alpha	N^b^	Test-retest (ICC)	FACT-An total/subscale	N^a^	Cronbach’s alpha	N^b^	Test-retest (ICC)
Physical functioning	188	0.90	77	0.83	Anemia subscale	190	0.92	76	0.83
Role-physical	193	0.93	76	0.69	Fatigue subscale	196	0.93	76	0.80
Vitality	193	0.82	75	0.64	Total FACT-An score	131	0.95	76	0.88
Role-Emotional	193	0.93	77	0.74	FACT-G score	133	0.91	76	0.86
Mental Health	193	0.81	77	0.75	Functional Well-Being	196	0.87	76	0.83
Social Functioning	194	0.76	77	0.69	Physical Well-Being subscale	196	0.84	76	0.83
Bodily Pain	194	0.88	77	0.74	Social Well-Being	137	0.80	76	0.76
General Health	192	0.69	77	0.75	Emotional Well-Being	193	0.79	76	0.72
Physical Component Summary score	–	0.92	76	0.81					
Mental Component Summary score	–	0.90	76	0.75					

Abbreviations: *ICC* intraclass correlation coefficient, *FACT-An* Functional Assessment of Cancer Therapy-Anemia, *FACT-G* Functional Assessment of Cancer Therapy-General, *SF-36* Medical Outcomes Survey Short Form-36

^a.^Number of subjects at Baseline

^b.^Number of subjects with average weekly Hb change within +/− 0.5 g/dL between Week 9 and Week 13/17 (study FGCL-4592-053/study FGCL-4592-041) and who have not received rescue therapy

### Convergent and known-groups validity

As expected, the SF-36 Vitality domain showed strong correlations with the FACT-An Fatigue and Anemia subscales (*r =* 0.76 and *r =* 0.77, respectively; Table 4). The correlations between the SF-36 and the FACT-An Anemia and Fatigue subscales generally were high.

**Table 4:** Convergent Validity – Correlations Between SF-36 and the FACT-An Fatigue and Anemia Subscales

SF-36 domain/Component Summary score	FACT-An subscale	
	Anemia	Fatigue
Physical functioning	0.63***	0.60***
Role-Physical	0.64***	0.61***
Vitality	0.77***	0.76***
Role-Emotional	0.53***	0.52***
Mental Health	0.54***	0.52***
Social Functioning	0.66***	0.67***
Bodily Pain	0.53***	0.53***
General Health	0.64***	0.61***
Mental Component Summary score	0.62***	0.62***
Physical Component Summary score	0.69***	0.66***

Spearman’s rank order correlation. *** *p <* 0.001

Abbreviations: *FACT-An* Functional Assessment of Cancer Therapy-Anemia, *SF-36* Medical Outcomes Survey Short Form-36

The correlations with Hb level were modest, particularly at baseline where the Hb range was limited by trial inclusion criteria (Table 4). The correlations with Hb level at Week 9 and 13/17 were similar: SF-36 Vitality correlated with Hb *r =* 0.28 (*p <* 0.001) and Role Physical score, *r =* 0.25 (*p <* 0.01), whilst the FACT-An Total had a correlation of *r =* 0.33 (*p <* 0.001), Anemia *r =* 0.28 (*p <* 0.001), and Fatigue *r =* 0.30 (*p <* 0.001).

For the assessment of known groups validity, a median split of the predefined SF-36 and FACT An scores were used, as described earlier in the methods section. Highly significant differences were found for all the key FACT-An and SF-36 domains: the FACT-An scores split by the SF-36 Physical Functioning domain were: FACT-Anemia subscale score (mean 46.4, [SD 13.9]) vs 61.6(12.2), the FACT Fatigue subscale 28.9(10.8) vs. 39.7(9.5), and the Total FACT-An score 118.3(28.8) vs 145.0(25.0), all *p* < 0.0001. Similarly, the corresponding median split using the SF-36 Vitality score and the SF-36 PCS scores were all highly significant for all the FACT-An domains FACT-Anemia., FACT Fatigue subscale and the FACT-An Total scores *p* < 0.0001. The SF-36 results showed the same pattern, i.e., the SF-36 scores split by the median FACT-An score showed large and significant differences (*p* < 0.0001) for Physical Functioning 32.3(9.5) vs 43.8(9.4); and Vitality 39.5(13.5) vs. 55.5(8.3). A split by FACT-Anemia and the FACT-An Fatigue scales showed the same pattern for the SF-36 Physical Functioning and Vitality domains and were all highly significant (*p* < 0.001).

For the SF-36, using a median split of Hb level to define the group, a significant difference was found for the Vitality domain score at baseline, *p <* 0.05 (Table 5). The FACT-An Total score, discriminated between groups based on a median split of Hb level. When comparing groups with an Hb level of < 11 vs ≥ 11 g/DL at Week 9, the FACT-An Total Score, and Anemia and Fatigue subscale produced significantly different scores (*p* < 0.05, *p* < 0.01, *P* < 0.01, respectively).

**Table 5:** Convergent Validity—Correlations^a^ of FACT-An and SF-36 Scales with Hb Level at Baseline, Week 9 and Week 13/17^b^

	Hb level		
	Baseline	Week 9	Week 13/17^b^
FACT-An Total/Subscale			
**Total FACT-An score**	0.20*	0.22*	0.33**
Total FACT-G score	0.22*	0.20*	0.34***
Physical well-being subscale	0.21*	0.17*	0.32**
Functional well-being subscale	0.22*	0.21*	0.29**
Social/family well-being subscale	0.18*	0.09	0.22*
Emotional well-being subscale	0.16*	0.20*	0.30**
Anemia subscale	0.14	0.22*	0.28*
Fatigue subscale	0.11	0.26**	0.30**
Trial outcome index	0.19*	0.22*	0.32**
SF-36v2 Domain/Component			
Physical Functioning	0.07	0.13	0.12
Role-physical	0.24**	0.23*	0.25*
Role-emotional	0.12	0.13	0.25*
Vitality	0.18*	0.23*	0.28**
Mental Health	0.23*	0.22*	0.27*
Social Functioning	0.10	0.12	0.31**
Bodily Pain	0.00	0.10	0.25*
General Health	0.17*	0.22*	0.26*
Mental Component Summary	0.20*	0.18*	0.30**
Physical Component Summary	0.10	0.19*	0.22*

^a.^Spearman’s rank order correlation. *p < 0.05; **p < 0.001; ***p < 0.001

^b.^Week 13 for NCT00671657; Week 17 for NCT01244763

### Ability to detect change

Both the SF-36 and Fact-An demonstrated the ability to detect change. Small improvements (relative to baseline) were observed in all SF-36 domain and Component Summary scores. Despite a high baseline Vitality score in both trials, sizeable gains in Vitality were observed in both trials. Larger gains were observed in the dialysis group, with a greater than three-point increase by Week 13 for the Physical Component Summary score, and the Physical functioning, Role-emotional, Role-physical, and Vitality domains. Only the Vitality change score achieved this cut-off in the non-dialysis group by Week 9 or Week 17. Large improvements in FACT-An Total and all FACT-An subscale scores (except for the Social Well-Being) were shown at Week 9, and were relatively stable by Week 13/17. When separating by trial, baseline mean scores were higher in the non-dialysis group for the FACT-An Total score and all subscale scores compared with the dialysis group, and gains were generally larger in dialysis group.

For FACT-An Anemia subscale-defined responders, significant differences between responders and non-responders were identified for all SF-36 domains and Component Summary change scores at both time points assessed. Similarly, significant differences were observed between responders and non-responders using FACT-An Fatigue-defined responders at Week 9 for all SF-36 scores, and for all except Physical functioning, General Health and PCS at Week 13/17.

Using SF-36 Vitality-defined responders, significant differences were identified for the FACT-An Total change score,, Anemia and Fatigue subscale change scores (*p <* 0.001) at Week 9 and Week 13/17 (*p <* 0.01). A similar pattern of results was identified using SF-36 Physical functioning-defined and Physical Component Summary-defined responders where significant differences were found for FACT-An Total, Anemia, and Fatigue subscale scores relative to baseline (*p <* 0.01).

Using Hb level to define responders, only the Fatigue subscale produced significant differences at Week 9 between responders and non-responders based on Hb level/change (*p <* 0.05), whilst at Weeks 11–13/15–17, significant differences were identified for the FACT-An Total score, the and Physical Well-Being subscales (*p <* 0.05). Significant differences were also identified in the Fatigue, subscale at Week 13/17 (*p <* 0.05); however, the non-responder sample size was particularly low (*n* = 19–30) in these analyses at the later time points.

### Important change scores

Table 6 shows the important change scores produced by each method for each SF-36 domain/Component Summary scores. The anchor-based methods were produced using relatively small sample sizes (*n* = 21–26). The estimates produced by linking were similar to the other anchor-based methods, and typically smaller than the distribution-based estimates. A central principle is that confidence in the estimate increases when the domains are more highly correlated with the anchor [41, 51, 52]. Of the anchors used, the FACT-An Anemia subscale had the strongest correlations with the target domains/Component Summary scores. For the Physical functioning, Vitality, and Physical Component Summary domains, the following important change score estimates are recommended: Physical functioning: 2–3 points; Vitality: 2–4 points; Physical Component Summary: 2–4 points.

**Table 6:** SF-36: Important Change Score Estimates Using Distribution-based, Anchor-based, and Linking Methods

SF-36 Domain/Component Summary score	1 SEM	0.5 SD	Anemia 4–7, Week 9	Anemia 4–7, Week 13/17	Fatigue 3–5, Week 9	Fatigue 3–5, Week 13/17	Linking FACT-An total	Linking anemia	Linking fatigue
Physical functioning	4.6	5.5	2.6	2.9	1.5	2.3	2.59	2.96	2.90
Role-Physical	6.2	5.6	1.7	2.4	1.0	0.7			
Vitality	7.2	6.0	5.7	3.1	3.9	1.0	2.80	3.20	3.13
Role-Emotional	7.0	6.9	2.9	−1.3	3.7	−2.4			
Mental Health	5.8	5.7	3.4	−0.2	3.2	0.9			
Social Functioning	6.4	5.8	1.4	1.0	0.5	−1.5			
Bodily Pain	5.7	5.6	2.1	−1.4	−0.2	−3.0			
General Health	4.8	4.8	3.1	−2.2	− 1.5	− 2.2			
Physical Component Summary score	4.2	4.7	2.0	1.5	−0.8	0.0	2.19	2.51	2.45
Mental Component Summary score	5.7	5.8	3.6	−0.5	3.8	−1.3			

Abbreviations: *FACT-An* Functional Assessment of Cancer Therapy-Anemia, *SD* standard deviation, *SEM* standard error of measurement, *SF-36* Medical Outcomes Survey Short Form-36

For the FACT-An scores, distribution-based estimates were typically larger than anchor-based estimates, with 0.5 SD estimates larger than one SEM estimates for all scores except the Social Well-Being subscale (Table 7).

**Table 7:** FACT-An: Important Change Score Estimates Using Distribution-based, anchor-based, and Linking Methods

FACT-An total/ subscale	1 SEM	0.5 SD	Vitality 3–4, Week 9	Vitality 3–4, Week 13/17	Physical functioning 2.8–4.2, Week 9	Physical functioning 2.8–4.2, Week 13/17	PCS 2–4, Week 9	PCS 2–4, Week 13/17	Linking vitality	Linking physical functioning	Linking PCS
Total	10.4	15.0	8.7	7.8	4.8	9.1	4.4	7.1	7.50	8.11	6.38
Anemia subscale	6.2	7.5	5.2	4.2	1.3	5.5	2.5	4.7	3.75	4.05	3.19
Fatigue subscale	5.2	5.7	4.0	3.2	2.1	3.9	2.1	3.6	2.88	3.11	2.45
General	6.4	8.5	3.6	3.6	3.5	3.6	1.9	2.4			
Physical Well-Being	2.3	2.7	0.7	1.5	−0.4	1.3	0.3	1.3			
Functional Well-Being	2.7	3.3	0.6	0.4	1.7	2.4	0.0	−0.4			
Social Well-Being	2.8	2.9	0.9	0.7	0.5	−0.5	0.9	1.7			
Emotional Well-Being	2.5	2.4	1.2	1.0	1.7	0.3	0.7	−0.2			

Abbreviations: *FACT-An* Functional Assessment of Cancer Therapy-Anemia, *PCS* Physical Component Summary, *SD* standard deviation, *SEM* standard error of measurement

One flaw of the anchor-based approach is frequent reliance on small sample sizes, as was the case in this study (*n* = 14–29), with the anchor range increased to 2.8 to 4.2 for the SF-36 Physical Component Summary change score (as no participants achieved a score change between these values at either time point). The linking estimates were similar to the other anchor-based methods.

## Discussion

Despite the relatively common use of the SF-36 in patients with anemia associated with CKD in clinical studies and clinical trials [6, 16–18, 20–23, 53, 54], the psychometric measurement properties of SF-36 have not previously been reported in this patient population. This study provides evidence of the reliability, validity, and responsiveness of the SF-36 measure, and the results support the use of the SF-36 to assess treatment efficacy in clinical trials in this patient population. For patients with anemia associated with CKD, tiredness, fatigue, and poor physical functioning each have a significant impact on HRQoL. Therefore, the Physical functioning and Vitality domains may prove particularly useful in assessing the major impacts of the treatment of anemia in these patients. For a more general assessment of physical impact, the Physical Component Summary can also be used.

Whereas the SF-36 was developed to measure overall HRQoL, the FACT-An Anemia subscale was developed specifically to capture the impact of anemia on HRQoL, with the shorter Fatigue and Anemia subscales capturing a major impact often noted in anemia. These subscales show particular promise for use as endpoints in the CKD anemia population for capturing the main patient health issues related to anemia; the strong correlations between the SF-36 and FACT-An domains scores further support the validity of these measures when used in a CKD population with anemia. The FACT-An Total score also captures these impacts and combines them with a general measure of HRQoL (FACT-G); thus, the FACT-An Total score is useful as an overall summary of HRQoL that can capture impairment to well-being resulting from anemia to describe the full impact of CKD with anemia.

When separated by trial, baseline mean scores were similar for the Fatigue and Anemia domains but slightly higher in the non-dialysis group for the FACT-An Total score and the other subscale scores. Moreover, these results highlight a somewhat greater impact on those patients with CKD receiving dialysis (i.e., those at a more severe stage of kidney failure), especially across social, functional, and emotional domains.

The link between anemia and fatigue, and the impact of CKD anemia on physical functioning, are each highlighted by baseline scores that were comparable with those found in patients with cancer [55]. These links are underlined by the observation that the correlation with the Hb level grew stronger over the duration of the treatment period. Similar correlations between Hb level and fatigue have been reported in patients with cancer [57].

The baseline PRO scores generally indicated a somewhat greater impact on patients with CKD receiving dialysis compared with the non-dialysis group, across social, functional, emotional domains and FACT-Anemia and Fatigue subscale scores. The improvement in these scores was also generally larger in the dialysis population. However, such a pattern was not shown for the SF-36 scores.

Reliability, assessed by internal consistency and test-retest correlation coefficients, was demonstrated for the SF-36 domain and summary scores (Cronbach’s alpha = 0.69–0.93; ICC = 0.64–0.83), and the FACT-An Total and all subscale scores (Cronbach’s alpha = 0.79–0.95; ICC = 0.72–0.88). As expected, high convergent validity was demonstrated for domains measuring similar concepts.

The strong correlation between the Vitality domain with each of the FACT-An subscales was encouraging. As the Vitality domain had the strongest relationship with the FACT-An Total score and Anemia and Fatigue subscales, extra consideration was given to the use of this anchor in determining an important change score. Consequently, the following important change score estimates are recommended: FACT-An Total: 6–9 points; Anemia: 3–5 points; Fatigue: 2–4 points.

The relatively high Vitality score in the present study is surprising given that a substantial degree of impairment was reported in previous studies of CKD patients with anemia [6, 7].

Known-groups validity was demonstrated for the selected key domains Physical Functioning, and Vitality with significant differences between groups defined using the FACT-An Total, Fatigue, and Anemia subscale scores. Similarly, the key FACT-An scores, i.e., the FACT-An Total, and Fatigue and Anemia subscale scores differed significantly when split by SF-36 scores. Correlations with Hb level were typically smaller, particularly at baseline, where the Hb level was constrained by the inclusion criteria. Hb level-defined groups produced mixed results, which is consistent with published data reporting that a modest relationship between PRO measures and Hb level has previously been shown, with fatigue and physical functioning measures demonstrating additional benefit beyond or in the absence of Hb level change [56–58].

In patients with cancer, the correlation between Hb level and measure of fatigue is moderate, albeit sufficiently high to support the use of Hb as a clinical anchor for validation purposes [41, 59]. Notably, Holzner et al. [58] identified differences in the Multi-dimensional Fatigue Inventory scores between patients with cancer and healthy subjects, despite both groups having a normal Hb range. Furthermore, patients with lung cancer grouped by FACT-Fatigue scale score (in tertiles) had no significant differences in Hb level but significant differences in physical functioning and psychological distress [56]. These findings highlight the importance of measures that capture concepts beyond Hb level change, as Hb is not the only indicator of disease burden in these patients (especially in light of improvement in PRO scores in the two trials). Specifically, in circumstances where objective clinical markers do not exclusively identify the treatment benefit, inclusion of validated PRO measures as additional measures of efficacy is important [15]. The low correlations with Hb level also indicate that in this study sample more factors influence quality of life than Hb alone. The important change score estimates were similar irrespective of whether anchor-based or linking-based approaches were used, which supports their validity [51]. The FACT-An and SF-36 estimates were also consistent with previously suggested values for these instruments [40, 41].

For use as an endpoint in clinical trials, a PRO instrument needs to be sensitive to changes in a patient’s condition. Although mixed results were identified using Hb level to define responders/non-responders in HRQoL measures, responsiveness was demonstrated using SF-36-defined responders/non-responders for all FACT-An scores. Coupled with a change in mean scores from baseline to Week 13/17 in both the SF-36 and FACT-An, these findings support the use of the SF-36 domains, Physical functioning and vitality, and the FACT-An scores for measuring efficacy from the patient perspective.

Our analysis has several limitations, including use of trial designs were similar, but not identical; however, the purpose of our research was to evaluate of the SF-36 and FACT-An questionnaires in patients with ESRD, which was unlikely to be affected by differences in trial duration or time of questionnaire administration. Both questionnaires were administered according to instructions provided. Prior research has suggested timing during a trial has no significant effect on responses [60]. The analyses were limited by the unavailability of clinical anchors that were not included in the trial, such as patient and clinician overall assessment of changes that would be useful in assessing the measurement performance, and in particular responsiveness to change of the two instruments. Although pooling the data of the two trials both increased the sample size and the range of severity, the trial samples differed in several sociodemographic (age, gender, and ethnicity) and clinical (CKD history and comorbid conditions) characteristics. However, even smaller sample sizes would be observed for the anchor-based important change score analyses had the data not been pooled. Whilst the results provide good evidence of the measurement properties of the SF-36 and the FACT-An, additional evidence of validity and responsiveness, in a larger sample, and using other variables in the analyses, is desirable. Increasing the sample size would provide more confidence in the estimation of anchor-based important change scores, as small sample sizes (such as in this study) are more vulnerable to individual extreme values distorting the mean. Hence, the MID estimates should be regarded as provisional with need for further corroboration in future trials. Using data derived in a clinical trial for validation purposes makes it possible to get an estimate of the magnitude of change observed. This is important for the assessment of responsiveness to change. By deriving additional data on the measurement properties in future trials further evidence can be provided. Despite its limitations, this study has demonstrated that the Physical functioning domain of the SF 36 in particular and the FACT-An Fatigue and Anemia subscales are useful measures for capturing important aspects of HRQoL in patients with CKD associated with suffering from anemia.

The results of this study provide further evidence of the reliability, validity and responsiveness of the SF-36 and FACT-An in patients with CKD receiving and not receiving dialysis. Both measures have already been included as endpoints in clinical trials for anemia associated with CKD [6, 16, 33].

## Conclusions

When evaluating the impact of anemia on patients with CKD, the SF-36 domains Vitality and Physical functioning scores, and the FACT-An Total, Fatigue, and Anemia domain subscales show good evidence of reliability, validity, and responsiveness. The modest relationship observed between Hb level and HRQoL highlights the importance of capturing HRQoL data, given patients with CKD treated for anemia experienced an improvement in FACT-An and SF-36 scores. Thus, the SF-36 and FACT-An questionnaires may be suitable for assessing the benefit of treatment beyond changes in Hb level in patients with anemia associated with CKD.

## Abbreviations

ANCOVA: Analysis of covariance
CKD: Chronic kidney disease
ESA: Erythropoiesis-stimulating agents
ESRD: End-stage renal disease
FACT-An: Functional assessment of cancer therapy-anemia
FACT-G: Functional assessment of cancer therapy-general
Hb: Hemoglobin
HIF: Hypoxia-inducing factor
HIF-PH: Hypoxia-inducing factor- prolyl hydroxylase
HRQoL: Health-related quality of life
ICC: Intraclass correlation coefficient
IV: Intravenous
MCS: Mental component summary
MID: Minimally important difference
PCS: Physical component summary
PRO: Patient-reported outcome
SD: Standard deviation
SEM: Standard error of measurement
SF-36: Medical outcomes survey short form-36
US: United States
